# A TREK‐1/AQP4/TRPA1/BDNF Signaling Axis Is Associated With Astrocytic Volume Transients, Synaptic Plasticity, and Spatial Memory

**DOI:** 10.1002/glia.70195

**Published:** 2026-07-15

**Authors:** Victor James Drew, Junsung Woo, Jung Moo Lee, Wuhyun Koh, Joungha Won, Jae‐Hun Lee, C. Justin Lee

**Affiliations:** ^1^ Center for Memory and Glioscience Institute for Basic Science (IBS) Daejeon Republic of Korea; ^2^ Department of Neuroscience, Division of Bio‐Medical Science & Technology Korea University of Science and Technology (UST) Seoul Republic of Korea

**Keywords:** AQP4, astrocytic volume transient, BDNF, spatial memory, synaptic plasticity, TREK‐1, TRPA1

## Abstract

Astrocytes, known for their support roles, are emerging as active participants in synaptic plasticity and cognitive functions. Astrocytes actively regulate synaptic plasticity and memory through dynamic volume transients. Our previous research identified several key molecules, including TREK‐1, TRPA1, and Best1 ion channels, as well as the gliotransmitter BDNF, as critical components of astrocytic volume transients. However, the precise mechanisms by which these volume transients influence synaptic plasticity and memory remain poorly understood. In this study, we investigate the roles of TREK‐1 and TRPA1 in astrocytic volume dynamics and their downstream effects. Our findings, based on intrinsic optical signal imaging, electrophysiology, and behavioral assays, support a model in which neuronal stimulation induces astrocytic swelling, initiated by K^+^ uptake through TREK‐1 channels and regulated by Ca^2+^ influx via TRPA1 channels. This swelling is closely associated with short‐ and long‐term potentiation (LTP), and exogenous BDNF restores LTP under conditions of calcium sequestration during astrocytic calcium clamping experiments. Disruption of ion channels associated with astrocytic volume transients leads to significant impairments in spatial memory, as demonstrated by deficits in object‐place recognition and passive avoidance tasks. Moreover, these channels contribute to the regulation of synaptic plasticity. These findings implicate astrocytic volume transients and BDNF as pivotal modulators of synaptic plasticity and memory, as well as potential therapeutic targets for addressing memory dysfunctions.

## Graphical abstract

- Astrocytic TREK‐1‐dependent volume transients contribute to hippocampal synaptic plasticity and memory formation.
- TRPA1‐mediated astrocytic calcium signaling is required for long‐term potentiation, while astrocytic BDNF is necessary and sufficient to support sustained synaptic plasticity.
- Together, the findings support a TREK‐1/AQP4/TRPA1/BDNF signaling framework linking astrocytic volume dynamics to cognitive function.

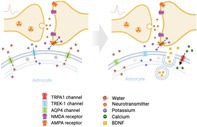

## 1 Introduction

Synaptic plasticity, the cellular mechanism underlying learning and memory, is a highly dynamic process orchestrated through intricate interactions between neurons and glial cells (De Pittà et al. 2016; Vernadakis 1996; Cornell et al. 2022). Once thought to only play a supportive role, astrocytes are increasingly recognized as active regulators of synaptic and circuit function. Astrocytic responses to neuronal activity involve transient volume changes driven by ion influx, accompanied by water entry mediated through water channels such as aquaporin‐4 (AQP4) (Woo et al. 2020). These volume transients have been implicated in modulating the synaptic microenvironment, potentially influencing plasticity and cognitive function (Chun et al. 2018).

Astrocytic volume transients are closely coupled to neuronal activity (Woo et al. 2020). Potassium uptake, facilitated by TWIK‐related K^+^ channel 1 (TREK‐1)‐containing two pore potassium (K2P) channels, triggers water influx through AQP4. This creates a swelling phenomenon that is followed by chloride efflux via bestrophin‐1 (Best1) anion channels, enabling volume recovery (Woo et al. 2020). These processes have been linked to synaptic potentiation and memory formation, as demonstrated by recent studies showing impaired long‐term potentiation (LTP) and memory in AQP4‐deficient models (Szu and Binder 2016). Furthermore, the dynamics of astrocytic swelling, captured through intrinsic optical signal (IOS) imaging (Woo et al. 2020), provide insight into their contribution to activity‐dependent extracellular ion and neurotransmitter homeostasis (Woo et al. 2019). Despite advances in understanding astrocytic contributions to plasticity, several critical gaps remain. While prior research has described the general roles of channels such as TREK‐1 and transient receptor potential ankyrin 1 (TRPA1), the specific interplay between astrocytic calcium signaling and the release of gliotransmitters like brain‐derived growth factor (BDNF) has not been fully delineated.

Among the gliotransmitters released, BDNF stands out due to its high expression levels in the brain and its well‐established effects on synaptic plasticity (Crozier et al. 2008; Leal et al. 2017). Pioneering research in the early 1990s demonstrated that a stimulation protocol capable of inducing LTP in the hippocampal CA1 region also increases BDNF mRNA expression in neurons (Patterson et al. 1992). Further investigations revealed that LTP is compromised in BDNF gene‐silencing mouse models but can be restored by reintroducing BDNF (Korte et al. 1995; Patterson et al. 1996). BDNF is associated with accelerated synaptogenesis in various subtypes of neurons, as well as improved memory function through activation of NMDA receptor signaling (Koshimizu et al. 2021; Song 2024). Interestingly, it has been demonstrated that astrocytic BDNF is also capable of modulating LTP and memory formation (Liu et al. 2022). Notably, the direct influence of astrocytic volume transients and corresponding calcium dynamics on both short‐term and long‐term plasticity, as well as their implications for spatial memory, remain an area of active investigation.

This study addresses these gaps by examining whether astrocytic calcium signaling associated with volume transient activation of mechanosensitive channels contributes to synaptic plasticity and memory, potentially through BDNF‐dependent mechanisms. Using a combination of electrophysiology, genetic manipulations, and behavioral assays, we investigate how astrocytic mechanisms impact synaptic potentiation and memory. By employing advanced tools such as tamoxifen‐inducible Cre‐lox systems (Hayashi and McMahon 2002) and pharmacological interventions, we provide new insights into how astrocytes support plasticity and memory through calcium‐dependent mechanisms and the targeted release of BDNF. Our findings highlight the contributions of astrocytic volume transients and calcium signaling to synaptic plasticity and memory and support a model linking astrocyte‐neuron interactions with memory formation.

## 2 Methods

### 2.1 Animals

Adult male mice (6–10 weeks old) were used in this study. Wildtype mice (C57BL/6; Jackson Laboratory, RRID: IMSR_JAX:000664), TRPA1 knockout (TRPA1 KO) mice (129 strain; Jackson Laboratory, RRID: IMSR_JAX:006401), GFAP‐GFP mice (Jackson Laboratory, RRID: ISMR_JAX:003257), and their respective wildtype littermates were included. Mice were housed in groups of 3–5 per cage under a standard 12‐h light/dark cycle with ad libitum access to food and water. All experimental procedures were approved by the Institutional Animal Care and Use Committee (IACUC) of the Institute for Basic Science (IBS, Daejeon, Korea; Protocol No. IBS‐22‐26) and conducted in accordance with institutional and national guidelines for animal care.

### 2.2 Immunohistochemistry

The mice were anesthetized with 1%–2% isoflurane, then underwent transcardiac perfusion with saline and 4% paraformaldehyde (PFA). The brain was removed and submerged into 4% PFA for post‐fixation at 4°C overnight. Subsequently, the brain was transferred into a 30% sucrose solution for cryoprotection and stored at 4°C. Brain tissue was frozen at −80°C and sectioned at 30 μm thickness across the coronal plane in a cryostat (CM1950, Leica Biosystems, Germany). For the brain tissue immunostaining, sections were incubated in blocking solution containing 0.3% Triton X‐100 (X100, Sigma‐Aldrich, USA), 4% donkey serum (GTX27475, Genetex, USA), and 0.1 M PBS. sections were then immunostained with anti‐GFAP (Millipore, AB5541, Burlington, MA, USA), anti‐TREK‐1 (Alomone Labs, APC‐047‐GP, Jerusalem, Israel), anti‐TRPA1 (Alomone Labs, ACC037, Jerusalem, Israel), and anti‐BDNF (Santa Cruz Biotechnology, sc‐20981, Dallas, TX, USA) primary antibodies in blocking solution at 4°C overnight at a ratio of 1:500. A list of antibody details is provided in Table S1. Following incubation with primary antibodies, sections were washed with 0.1 M PBS three times for 10 min, then secondary antibodies (Jackson Immuno Research Laboratories, USA). with corresponding fluorescence were applied in fresh blocking solution for 2 h to the brain sections. Following incubation with secondary antibodies, sections were washed with 0.1 M PBS three times for 10 min. Next, DAPI (62,248, Thermo Fisher Scientific, USA) was added at a ratio of 1:1000 during the second wash to stain the nucleus. Brain sections were mounted on silane‐coated slide glass (5116‐20F, Muto Pure Chemicals, Japan) with fluorescence mounting medium (S3023, Dako, Denmark). Fluorescent images were acquired using a Zeiss LSM900 confocal microscope with Z‐stacks collected in 1 μm intervals.

### 2.3 Image Quantification

Fluorescent images obtained from confocal microscopy were analyzed using ImageJ Fiji (NIH, USA). To quantify TREK‐1, TRPA1, and BDNF immunoreactive intensity, all images were z‐stacked with maximum‐intensity by pixel, and set at the same brightness and contrast. The regions of interest (ROIs) were set by calculating the area of GFAP with the same threshold setting throughout the whole image. Intensity in each ROI was analyzed in 8‐bit images. To quantify the astrocytic TREK‐1, TRPA1, or BDNF expression levels in the CA1 hippocampus, the GFAP‐positive, TREK‐1‐, TRPA1‐, or BDNF‐positive regions were selected for ROIs. Intensity in astrocyte‐shaped ROIs was calculated.

### 2.4 Slice Preparation

Hippocampal slices were prepared as described previously (Woo et al. 2019). Briefly, mice were anesthetized with 2%–4% isoflurane inhalation and decapitated while under anesthesia. The brains were rapidly extracted and placed in ice‐cold, oxygenated (95% O_2_, 5% CO_2_) high‐Mg^2+^ dissection buffer containing the following (in mM): 130 NaCl, 24 NaHCO_3_, 3.5 KCl, 1.25 NaH_2_PO_4_, 1 CaCl_2_, 3 MgCl_2_, and 10 glucose (pH 7.4). Transverse hippocampal slices (300 μm thick) were obtained using a D.S.K. Linear Slicer Pro 7 (Dosaka EM Co. Ltd., Japan). The slices were incubated in the high‐Mg^2+^ dissection buffer at room temperature for at least 1 h to allow for recovery. Subsequently, the buffer was replaced with oxygenated artificial cerebrospinal fluid (aCSF) containing (in mM): 130 NaCl, 24 NaHCO₃, 3.5 KCl, 1.25 NaH_2_PO_4_, 1.5 CaCl_2_, 1.5 MgCl_2_, and 10 glucose (pH 7.4), and the slices were further recovered before use in IOS or electrophysiological recording experiments.

### 2.5 Intrinsic Optical Signal Recording

IOS recording was performed as previously described (Woo et al. 2020; Woo et al. 2019; Woo, Kim, et al. 2018). Briefly, submerged hippocampal slices were transilluminated using a controlled infrared (IR) light source equipped with an optical filter (775 nm wavelength, Omega Filters). Images were captured using an Olympus BX50WI microscope paired with a Hamamatsu ORCA‐R2 digital CCD camera. Imaging was focused on the stratum radiatum of the hippocampal CA1 region. A series of 80 images per second was acquired following a 20 Hz, 1‐s electrical stimulation. The relative change in transmittance (Δ*T*/*T*) was normalized to the baseline, defined as the average transmittance of the five pre‐stimulation images. The decay of the IOS was calculated by averaging the last 10 s of the response, with responses normalized to the peak value.

### 2.6 Whole‐Cell Recordings of Long‐Term Potentiation

Whole‐cell patch‐clamp recordings were conducted on pyramidal neurons within the hippocampal CA1 using a Multiclamp 700B amplifier (Molecular Devices, Union City, NJ, USA). Borosilicate glass patch pipettes (resistance: 5–8 MΩ) were filled with an intracellular solution comprising (in mM): 126 potassium gluconate, 5 HEPES, 0.5 MgCl₂, and 10 BAPTA, with the pH adjusted to 7.3 using KOH. For experiments requiring astrocyte labeling, Sulforhodamine 101 (SR101) was loaded into the pipette. Positive pressure was maintained on the pipettes during advancement through the tissue. A concentric bipolar stimulation electrode (FHC, Bowdoin, ME, USA) was positioned approximately 400 μm from the patched astrocyte. Signals were low‐pass filtered at 2 kHz, digitized at 10 kHz using a Digidata 1322A digitizer (Molecular Devices), and analyzed with pClamp 10.2 software (Molecular Devices). Evoked excitatory postsynaptic current (eEPSC) recordings were conducted as previously described (Woo et al. 2019). Briefly, eEPSCs in the CA1 stratum radiatum were induced through Schaffer collateral stimulation with a concentric bipolar electrode. Recording pipettes (resistance: 1–3 MΩ) were filled with artificial cerebrospinal fluid (aCSF). The amplitude of eEPSCs was measured and used for subsequent analyses. LTP was assessed at the CA3‐CA1 synaptic pathway in the hippocampus using whole‐cell patch‐clamp recordings from CA1 pyramidal neurons. A stimulating electrode was positioned along the Schaffer collateral fibers to evoke eEPSCs at 0.1 Hz. LTP was induced using a theta‐burst stimulation (TBS) protocol, which consisted of 10 trains of four half‐maximal stimuli delivered at 100 Hz, with a 200 ms inter‐train interval, while maintaining the neuron at a holding potential of 0 mV. Baseline eEPSCs were recorded for 5 min before TBS to ensure stability. To minimize run‐up effects and optimize stimulation conditions, a test pulse (0.1 Hz) was applied during the giga‐seal configuration for approximately 10 min, allowing fiber stabilization. Stimulation intensity was calibrated to 150%–200% of the action potential threshold during this phase. LTP protocols were initiated within 10 min of achieving whole‐cell configuration to reduce potential washout effects from the internal pipette solution. eEPSCs were recorded every 10 s with neurons clamped at a holding potential of −60 mV. Recording pipettes (resistance: 6–8 MΩ) were filled with an intracellular solution containing (in mM): 135 cesium methanesulfonate, 8 NaCl, 10 HEPES, 0.25 EGTA, 1 Mg‐ATP, and 0.25 Na‐GTP, adjusted to pH 7.2 with NaOH and an osmolarity of 290 mOsm. The eEPSC amplitudes were normalized to the average baseline amplitude for subsequent analysis.

### 2.7 Passive Avoidance Test

The passive avoidance test was conducted to evaluate associative learning and memory (Ader et al. 1972). Mice (6–7 weeks old) were placed in the light compartment of a two‐chamber apparatus and allowed to explore freely for 60 s. After this habituation period, the door to the dark compartment was raised, and the mice were allowed to explore both compartments freely. The latency to enter the dark compartment with all four paws was recorded as the baseline latency. On Day 2 (training session), the mice were again placed in the light compartment, and the latency to enter the dark compartment was recorded. Upon full entry into the dark compartment, a foot shock (0.5 mA, 2‐s duration) was delivered 3 s after the door closed. Mice were returned to their home cages 30 s after the foot shock. On the test day (24 h post‐training), mice were placed back in the light compartment. After 5 s, the door to the dark compartment was lifted, and the latency to enter the dark compartment was recorded to assess memory retention.

### 2.8 Object‐Place Recognition Test

Behavioral testing was conducted in a gray Plexiglass box (30 × 30 × 40 cm) equipped with distinct visual cues. Animals underwent 3 days of handling before testing. To habituate to the environment, animals were placed in the arena for 10 min on two consecutive days. On the third day, during the training session, animals were exposed to two identical objects placed within the arena for 10 min. In the test session, conducted 1 h later, animals were re‐exposed to the same arena for 5 min. One of the objects was displaced (Displaced Object), while the other remained stationary (Stationary Object). All sessions were videotaped for subsequent analysis. Exploration behavior was assessed by experimenters blinded to the experimental groups. Exploration time was defined as the duration during which the animal oriented its head toward an object within a distance of less than 1 cm. Performance measurements were expressed as exploration ratios, calculated as follows:

$$\begin{array}{c} \text{Stationary Exploration}\;\left(\%\right)\\ =\frac{\left(\text{Time exploring Stationary Object}\right)}{\left(\text{Time exploring Stationary Object}+\text{Time exploring Displaced Object}\right)}\\ \times 100 \end{array}$$

$$\begin{array}{c} \text{Displacement Exploration}\;\left(\%\right)\\ =\frac{\left(\text{Time exploring Displaced Object}\right)}{\left(\text{Time exploring Stationary Object}+\text{Time exploring Displaced Object}\right)}\\ \times 100 \end{array}$$

### 2.9 Stereotaxic Surgery and Viral Injection

Mice (7–8 weeks old) were anesthetized with 3%–5% isoflurane inhalation and positioned in a stereotaxic frame. During surgical procedures, the isoflurane concentration was reduced to 1%–3%. Each surgery was completed within 1 h per mouse. Viral constructs, including an astrocyte membrane‐targeting calcium indicator (AAV9‐GFAP‐LCK‐GCamp6f), or pSicoR lentivirus containing shRNA targeting TREK‐1 (5′‐GCGTGGAGATCTACGACAAGT‐3′) (pSicoR‐TREK‐1‐shRNA‐mCherry) and BDNF (5′‐GGTGATGCTCAGCAGTCAAGT‐3′) (pSicoR‐BDNF‐shRNA‐mCherry), as well as scrambled controls (pSicoR‐scrambled‐shRNA‐mCherry), were loaded into a microdispenser (VWR, Radnor, PA, USA) for bilateral injection into the hippocampal CA1 region (−1.7 mm AP, ±1.7 mm ML, 1.8 mm DV from the dura). A total volume of 0.5 μL per hemisphere was injected at a rate of 0.1 μL/min using a 25 μL syringe connected to a syringe pump (KD Scientific, USA). Lentiviral constructs were produced at the Virus Facility of the Institute for Basic Science (IBS, Daejeon, Republic of Korea). Viral titers were determined by real‐time quantitative PCR using primers targeting the ITR sequence (qPCR Lentivirus Titer Kit; Applied Biological Materials Inc., Richmond, BC, Canada; Cat. No. LV900). The viral titers were as follows: pSicoR‐scrambled‐shRNA‐mCherry, 3.34 × 10 (Woo et al. 2019) infectious units (IU)/mL; pSicoR‐shTREK‐1‐mCherry, 2.56 × 10^8^ IU/mL; and pSicoR‐shBDNF‐mCherry, 2.94 × 10^8^ IU/mL. shRNA vectors were previously validated for effective knockdown of TREK‐1 and BDNF in astrocytes (Woo et al. 2020; Yoo et al. 2016) and were reconfirmed for this study (Figure S1). To achieve glial‐specific gene rescue, the target shRNA cassette was flanked by loxP sites to enable Cre‐loxP recombination, which excised the shRNA cassette and inactivated the target shRNA (Ventura et al. 2004). Selective retention of target gene expression in glial cells was achieved by injecting the virus into transgenic mouse lines that conditionally express Cre recombinase in glial cells, including hGFAP‐CreERT2 and ALDH1/1‐CreERT2 (Lee et al. 2010; Woo et al. 2012). For experiments involving BDNF rescue, an ALDH1/1‐CreERT2 mouse line crossed with BDNF floxed (BDNF fl/fl) mice was used. CreERT2 activation was induced by intraperitoneal administration of tamoxifen (1 mg dissolved in sunflower oil) or sunflower oil as a control, administered once daily for seven consecutive days prior to shRNA injection. Unless otherwise specified, electrophysiological and behavioral experiments were performed 7 days following viral injection. This interval was selected based on previous validation studies demonstrating robust shRNA expression and effective knockdown of TREK‐1 and BDNF within this time window (Woo et al. 2020; Yoo et al. 2016). The same post‐injection interval was used across lentiviral experimental groups to ensure consistency between electrophysiological and behavioral analyses. All experiments, including behavioral analyses and electrophysiological recordings, were performed under blinded conditions.

### 2.10 Chemicals

#### 2.10.1 Calcium Chelation with BAPTA

BAPTA, a high‐affinity calcium chelator, was utilized to disrupt intracellular calcium dynamics in astrocytes. For experiments requiring calcium chelation, BAPTA was incorporated into the intracellular solutions of glass micropipettes. The specific compositions of the internal solutions were as follows: (concentrations in mM): (De Pittà et al. 2016) 10 potassium BAPTA and 68 potassium gluconate, (Vernadakis 1996) 40–60 potassium BAPTA, or (Cornell et al. 2022) 0.1–1 potassium EGTA and 108 potassium gluconate. The osmolarity of all intracellular solutions was adjusted to 285 mOsmol using appropriate osmolarity correction methods, and the pH was titrated to 7.2 using potassium hydroxide (KOH). These preparations ensured compatibility with intracellular physiology during patch‐clamp recordings.

#### 2.10.2 Astrocyte Identification with SR101

SR101 (1 μmol/L; product code S7635, Sigma‐Aldrich, St. Louis, MO, USA), a red fluorescent xanthene derivative, was employed to facilitate astrocyte identification. SR101 was included in the intracellular solution of the patch pipette at the specified concentration. Following the successful patching of an astrocyte, SR101 was allowed to diffuse through the astrocytic syncytium via gap junctions. This diffusion enabled the fluorescent labeling and subsequent visualization of the astrocyte network, thereby indicating astrocyte identity in experimental preparations.

### 2.11 Statistical Analysis

Data are presented as means ± standard error mean (S.E.M.). Statistical analyses and graphing were performed using Prism 7 (GraphPad, San Jose, CA, USA) and SigmaPlot (Systat Software, San Jose, CA, USA). For comparisons between two groups, statistical significance was determined using a two‐tailed Student's *t*‐test. For comparisons involving multiple groups, one‐way analysis of variance (ANOVA) or two‐way repeated measures ANOVA was employed. When significant interactions were detected among multiple groups, post hoc analyses were conducted using Bonferroni or Tukey tests. An experiment‐specific description of statistical analyses, post hoc tests, and assumptions can be found in Table S2.

### 2.12 Artificial Intelligence (AI)‐Assisted Language Refinement and Editing

AI‐based language tool ChatGPT 5.5 was used solely for the purpose of language refinement, which included sentence restructuring and grammar corrections of manuscript text. AI tools were not used for data or image generation, data or statistical analysis, interpretation of experimental findings, or autonomous scientific writing. All scientific content, including the experimental design, data interpretation, and conclusions, was performed and verified by the authors. All AI‐assisted outputs were critically reviewed, edited, and validated by the authors prior to inclusion in the manuscript.

## 3 Results

### 3.1 Astrocytic TREK‐1 Is Associated With Synaptic Plasticity and Spatial Memory

**FIGURE 1:**
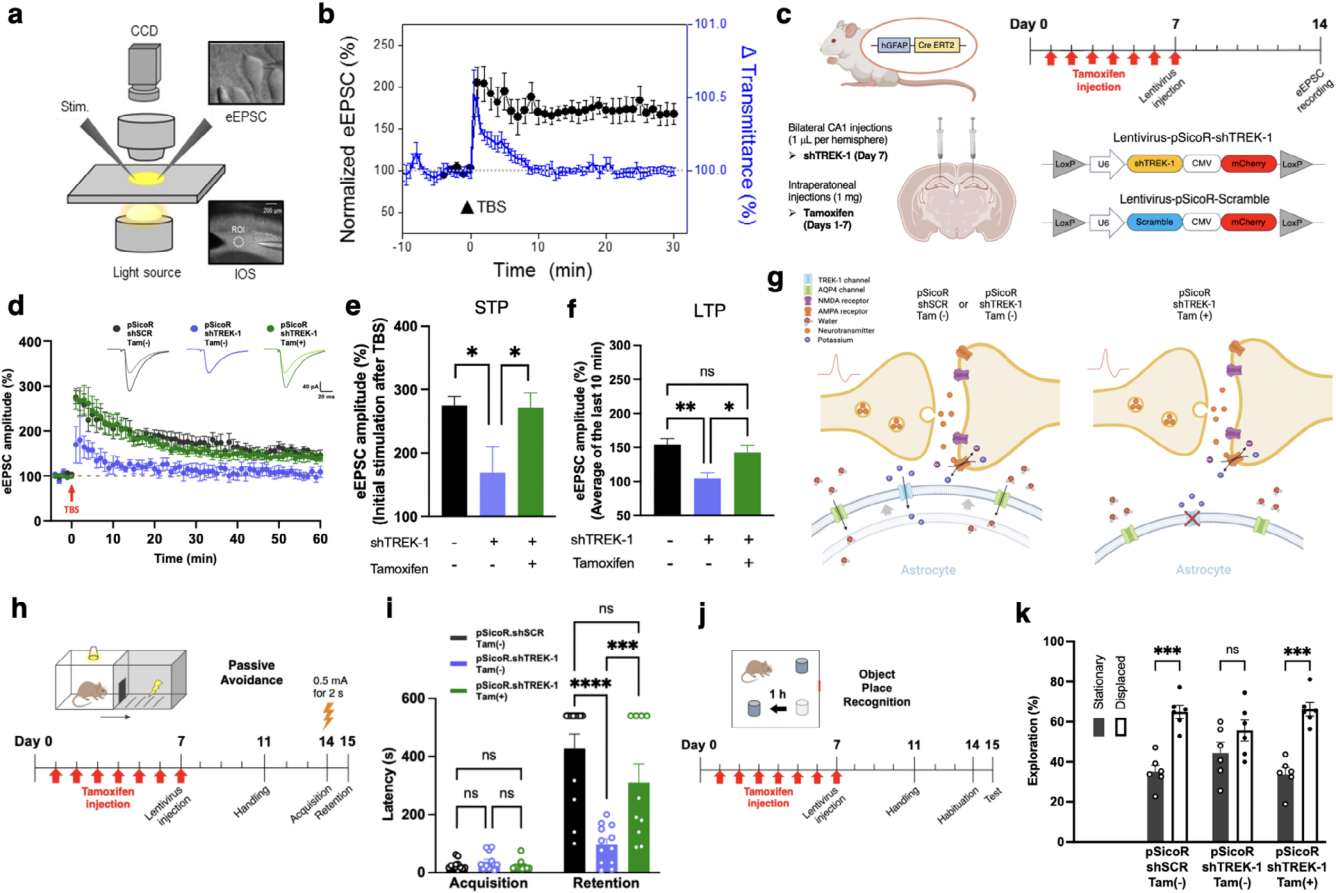
Astrocytic TREK‐1 is essential for synaptic plasticity and spatial memory. (a) Schematic representation of the IOS imaging and electrophysiological setup. The CA1 region of hippocampal slices was illuminated using an infrared light source, and changes in transmittance (Δ*T*/*T*) were recorded. eEPSCs were simultaneously measured using whole‐cell patch‐clamp techniques. (b) IOS and eEPSC responses to TBS. TBS induces a rapid transient decrease in IOS (blue) corresponding to a significant increase in normalized eEPSC amplitudes (black). These changes reflect activity‐dependent synaptic modifications. Data represent mean ± SEM. (c) Experimental design for astrocyte‐specific TREK‐1 knockdown and rescue. GFAP‐Cre.ERT2 transgenic mice received bilateral CA1 injections of lentivirus encoding shTREK‐1 or scrambled shRNA (control). Tamoxifen was administered for 7 days to induce astrocyte‐specific TREK‐1 knockdown, followed by electrophysiological assays 7 days post‐injection. shSCR, *n* = 5; shTREK‐1 Tam(−), *n* = 6; shTREK‐1 Tam(+), *n* = 7. (d) Representative traces and quantification of eEPSC amplitude during 60 min post‐TBS. TREK‐1‐deficient mice (pSicoR‐shTREK‐1 Tam(−)) exhibit significantly impaired short‐term potentiation (STP) and long‐term potentiation (LTP) compared to controls (pSicoR‐shSCR Tam(−)). TREK‐1 rescue (pSicoR‐shTREK‐1 Tam(+)) restores normal synaptic plasticity. (e, f) Quantification of eEPSC amplitudes immediately after TBS (e) and at the final 10 min of recording (f). TREK‐1 knockdown reduces synaptic potentiation, which is rescued by TREK‐1 re‐expression. Statistical comparisons for panels (e) and (f): One‐way ANOVA with Tukey post hoc test (*p < 0.05; **p < 0.01; ns = not significant). (g) Mechanistic model for TREK‐1‐mediated regulation of astrocytic volume transient. TREK‐1 channels mediate potassium influx during synaptic activity, creating osmotic gradients that facilitate water influx via AQP4. The resulting astrocytic swelling may mechanically modulate synapse function. In TREK‐1‐deficient astrocytes, impaired potassium buffering disrupts these processes, reducing synaptic efficacy. (h–k) Behavioral consequences of TREK‐1 deficiency. (h) Passive avoidance task timeline. shSCR Tam(−), *n* = 12; shTREK‐1 Tam(−), *n* = 12; shTREK‐1 Tam(+), *n* = 10. (i) TREK‐1‐deficient mice exhibit reduced retention latency, indicative of long‐term memory deficits. (j) Object‐place recognition test timeline. All groups, *n* = 6. (k) TREK‐1‐deficient mice display reduced exploration of displaced objects, indicating spatial memory impairments. TREK‐1 rescue restores normal performance. Statistical comparisons for panels (i) and (k): Two‐way repeated measures ANOVA with Bonferroni post hoc tests (***p < 0.001; ****p < 0.0001; ns = not significant). Data represent mean ± SEM.

To investigate the relationship between astrocytic volume transients and synaptic plasticity, we employed the IOS imaging technique (Woo et al. 2019; Woo, Kim, et al. 2018; MacVicar and Hochman 1991; Macvicar et al. 2002) to indirectly observe transient volume changes in real‐time in hippocampal slices by detecting light transmittance during intense neuronal activity in 2‐month‐old C57BL/6 (B6) mice. The neuronal activity‐induced volume transients were defined as the astrocytic volume change occurring within 1 min of intense neuronal activity. IOS imaging and electrophysiological recordings were conducted simultaneously in CA1 neurons of the hippocampus (Figure 1a). Stimulating electrodes were positioned in the stratum radiatum of the CA3‐CA1 Schaffer collateral pathway, enabling precise correlation between light transmittance changes and synaptic plasticity. LTP was induced using a theta‐TBS protocol, which consisted of 10 trains of four half‐maximal stimuli delivered at 100 Hz, with a 200 ms inter‐train interval, while maintaining the neuron at a holding potential of 0 mV. Baseline eEPSCs were recorded for 5 min before TBS to ensure stability. TBS elicited a rapid increase in light transmittance, accompanied by a simultaneous increase in normalized amplitude of eEPSC, occurring within seconds (Figure 1b). During short‐term potentiation (STP), the normalized amplitude of eEPSC exhibited a decline (exponential decay kinetics) over 10 min before stabilizing into LTP at over 150% of baseline, while light transmittance returned to baseline within the same timeframe. These results indicate that the IOS changes mirror the dynamics of STP, with both signals showing temporally correlated reductions following TBS. Although IOS imaging measures global changes in tissue transmittance, multiple pharmacological studies have demonstrated that the IOS response in hippocampal slices is primarily attributable to astrocytic volume changes. In particular, Woo et al. (2019) provided direct evidence that IOS changes evoked by neuronal stimulation at 20 Hz are abolished by genetic silencing of astrocytic AQP4, indicating that IOS under these conditions reflect water influx‐driven astrocytic swelling (Woo et al. 2019). The decay of IOS is likewise mediated by astrocytic Best1 channels, suggesting both the rise and recovery phases of IOS reflect astrocytic volume dynamics (Woo et al. 2020). These findings collectively support the interpretation that IOS, under the specific stimulation parameters employed in our study, serves as a robust proxy for astrocytic volume transients.

To explore the role of astrocytic TREK‐1 in synaptic plasticity, we employed GFAP‐CreERT2 transgenic mice for astrocyte‐specific gene silencing using a tamoxifen‐inducible Cre‐lox system (Figure 1c). The efficacy of TREK‐1 shRNA and tamoxifen toward the respective knockdown and rescue of astrocyte specific TREK‐1 in GFAP‐CreERT2 mice has been previously demonstrated and knockdown of TREK‐1 channels has been previously shown to disrupt astrocytic volume transients in response to neuronal activity (Woo et al. 2020; Woo, Bae, et al. 2018). Here, mice received tamoxifen injections for 7 days, followed by bilateral CA1 injections of lentivirus encoding either shTREK‐1_mCherry or control shSCR_mCherry. Seven days after viral delivery, hippocampal slices were prepared for electrophysiological recordings. In control mice (pSicoR‐shSCR Tam (−)), TBS reliably induced a robust STP and LTP, evidenced by sustained increases in normalized eEPSC amplitudes (Figure 1d,f). In contrast, TREK‐1 gene‐silencing (pSicoR‐shTREK‐1 Tam (−)) significantly impaired both STP and LTP, as shown by a diminished eEPSC peak and a gradual return toward baseline. Importantly, astrocytic TREK‐1 rescue (pSicoR‐shTREK‐1 Tam (+)) restored STP and LTP, with normalized eEPSC amplitudes comparable to controls (Figure 1e,f). Quantitative analysis showed a significant reduction in post‐TBS eEPSC amplitudes in TREK‐1‐deficient mice compared to both control and astrocytic TREK‐1‐rescue groups, supporting a functional contribution of astrocytic TREK‐1‐associated signaling to synaptic plasticity.

A mechanistic model for astrocytic TREK‐1 function in synaptic plasticity involves potassium buffering during synaptic activity (Figure 1g). TREK‐1 channels mediate astrocytic potassium influx, preventing extracellular potassium accumulation that could impair neuronal function. This influx generates an osmotic gradient, facilitating water influx through astrocytic aquaporin‐4 (AQP4) channels. The resulting astrocytic swelling may influence synaptic plasticity through mechanical signaling at neuron‐astrocyte interfaces. In TREK‐1‐deficient astrocytes, impaired potassium buffering likely disrupts these processes, leading to reduced synaptic plasticity (Lu et al. 2014).

We further examined the behavioral consequences of TREK‐1 deficiency using passive avoidance and object place recognition tests (Figure 1h,j). TREK‐1‐deficient mice exhibited impaired retention in the passive avoidance task compared to controls, as reflected by significantly reduced latency during the retention phase (Figure 1i). Similarly, in the object place recognition task, TREK‐1‐deficient mice showed reduced exploration of displaced objects, indicating deficits in spatial memory (Figure 1k). Astrocytic TREK‐1 rescue restored performance in both tasks to levels comparable to controls, suggesting a critical function of astrocytic TREK‐1 in spatial memory formation. Together, these findings support an important association between TREK‐1‐dependent astrocytic mechanisms and synaptic plasticity and memory, facilitated through its TREK‐1‐mediated volume transient function during potassium buffering.

### 3.2 TRPA1 Deficiency Is Associated With Impaired Astrocytic Calcium Signaling, Synaptic Plasticity and Memory Formation

**FIGURE 2:**
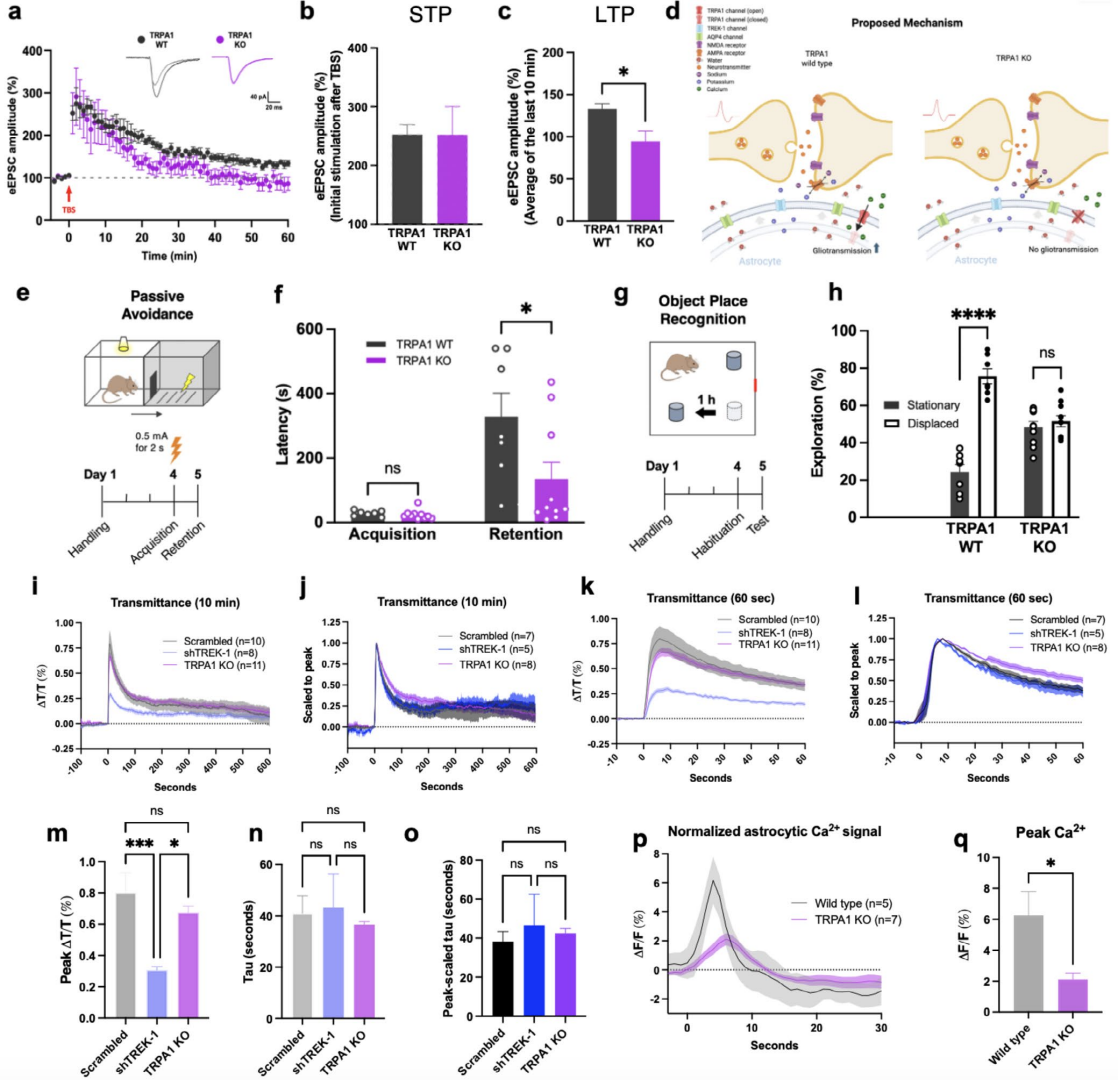
Astrocytic TRPA1 is essential for synaptic plasticity and spatial memory. (a) Time‐course of eEPSC amplitudes recorded from hippocampal CA1 neurons following TBS. TRPA1 WT mice exhibit sustained LTP over 60 min, whereas TRPA1 KO mice show impaired LTP with eEPSC amplitudes returning to baseline. Representative traces are shown above the graph for TRPA1 WT, *n* = 6 (black) and TRPA1 KO, *n* = 6 (purple). Data are presented as mean ± SEM. (b, c) Quantification of eEPSC amplitudes immediately after TBS (b) and during the final 10 min of recording (c). TRPA1 KO mice exhibit significantly reduced eEPSC amplitudes compared to TRPA1 WT mice, indicating a critical role for TRPA1 in maintaining LTP. Statistical analysis for panels (b) and (c): Unpaired two‐tailed Student's t‐test (*p < 0.05; ns = not significant). (d) Proposed model illustrating a potential role of TRPA1 in astrocyte‐neuron signaling. In TRPA1 WT astrocytes, potassium and water influx during synaptic activity activate mechanosensitive TRPA1 channels, leading to calcium influx and gliotransmitter release. This enhances synaptic efficacy. In TRPA1 KO astrocytes, the absence of calcium signaling prevents gliotransmitter release, impairing synaptic plasticity. (e–h) Behavioral analyses reveal deficits in long‐term memory in TRPA1 KO mice. (e) Passive avoidance test timeline. TRPA1 WT, *n* = 7; TRPA1 KO, *n* = 10. (f) TRPA1 KO mice exhibit significantly reduced retention latency compared to TRPA1 WT mice, indicating impaired associative memory. (g) Object‐place recognition test timeline. TRPA1 WT, *n* = 7; TRPA1 KO, *n* = 8. (h) TRPA1 KO mice display reduced exploration of displaced objects, reflecting deficits in spatial memory. Statistical analysis for panels (g) and (h): Two‐way repeated measures ANOVA with Bonferroni post hoc test (*p < 0.05; ****p < 0.0001; ns, not significant). Data are presented as mean ± SEM. (i) Normalized IOS (ΔT/T) responses recorded from the hippocampal CA1 stratum radiatum over a 10‐min period following TBS in scrambled control, astrocytic shTREK‐1, and TRPA1 KO mice. Astrocytic TREK‐1 knockdown resulted in a reduction in peak IOS amplitude, whereas TRPA1 KO mice exhibited IOS responses comparable in magnitude to scrambled controls. (j) Peak‐scaled IOS traces corresponding to the recordings shown in (i), scaled to their respective peak amplitudes to enable direct comparison of rise and decay kinetics across experimental groups. (k) Normalized IOS responses focusing on the first 60 s following TBS, highlighting early phase volume transient dynamics in scrambled, shTREK‐1, and TRPA1 KO mice. (l) Peak‐scaled IOS traces corresponding to the early response period shown in (k), allowing comparison of initial IOS kinetics independent of peak magnitude. (m) Quantification of peak IOS amplitude (ΔT/T) following TBS. Astrocytic TREK‐1 knockdown significantly reduced peak IOS amplitude, whereas TRPA1 deletion did not alter peak IOS magnitude relative to scrambled controls. (n, o) Quantification of IOS decay kinetics expressed as tau values normalized to the pre‐stimulation baseline (n) and following peak‐scaling (o). No significant differences in IOS decay kinetics were observed between scrambled and TRPA1 KO mice, indicating preserved volume transient recovery in the absence of TRPA1. Statistical analysis for panels (m–o): One‐way ANOVA with Tukey post hoc test (*p < 0.05; ***p < 0.001; ****p < 0.0001; ns, not significant). Data are presented as mean ± SEM. (p) Normalized astrocytic calcium responses following TBS, measured using an astrocyte membrane‐targeted calcium indicator (AAV9‐GFAP‐LCK‐GCaMP6f). TRPA1 WT astrocytes exhibited robust stimulus‐evoked calcium elevations, whereas TRPA1 KO astrocytes showed significantly attenuated calcium responses. (q) Quantification of peak astrocytic calcium signal amplitude following TBS, demonstrating a significant reduction in calcium responses in TRPA1 KO mice compared with WT controls. Statistical analysis for panel (q): Unpaired two‐tailed Student's *t*‐test (*p < 0.05). Data are presented as mean ± SEM.

To explore the potential role of astrocytic volume transient‐induced calcium signaling in synaptic plasticity and memory, we investigated TRPA1 channels due to their mechanosensitive characteristics and function in astrocytic calcium signaling. The role of astrocytic TRPA1 in synaptic plasticity was examined through electrophysiological recordings of eEPSCs in hippocampal slices obtained from TRPA1 WT and KO mice. The efficacy of the TRPA1 KO model has been previously established (Woo et al. 2020). Following TBS, TRPA1 WT mice displayed strong LTP, evident as a sustained elevation (Figure 2a). In contrast, TRPA1 KO mice showed markedly impaired LTP compared to TRPA1 WT mice, with eEPSC amplitudes declining to baseline by the end of the recording period (Figure 2a). STP was assessed and found to remain unchanged between TRPA1 KO and WT mice (Figure 2b), as indicated by comparable eEPSC amplitudes immediately following TBS. This finding suggests that TRPA1 deficiency preferentially affects mechanisms associated with long‐term synaptic plasticity rather than STP. Quantitative analysis further demonstrated the significant differences in LTP between TRPA1 WT and KO mice. eEPSC amplitudes were significantly lower in TRPA1 KO mice compared to TRPA1 WT mice during the sustained phase of LTP (Figure 2c). These results are consistent with the hypothesis that astrocytic membrane stretching during volume transient processes contributes to TRPA1‐associated calcium signaling involved in LTP. Furthermore, the reduction in eEPSC amplitudes observed in LTP but not in STP in TRPA1 KO mice suggests that STP is upstream of the involvement of TRPA1 in synaptic plasticity, emphasizing a specific role for TRPA1 in the later stages of synaptic potentiation.

Mechanistically, our findings are consistent with a model in which TRPA1‐dependent calcium signaling contributes to synaptic plasticity following neuronal activity‐induced astrocytic swelling. Potassium and water influx in astrocytes during synaptic activity activate TRPA1 channels via mechanosensitive gating, initiating calcium influx and subsequent gliotransmitter release (Figure 2d). Prior research has demonstrated that TRPA1 activation elicits calcium influx in astrocytes and triggers gliotransmission (Shigetomi et al. 2013; Woo et al. 2020). In light of these findings, the impaired LTP observed in TRPA1 KO mice in our experiments is consistent with a disruption of this calcium‐dependent astrocyte‐neuron signaling pathway. Our data support an association between TRPA1‐dependent calcium signaling and normal LTP, likely through its role in mediating astrocytic responses to volume transients and enabling gliotransmitter release.

The behavioral relevance of TRPA1 deficiency was evaluated using the passive avoidance and object place recognition tasks. TRPA1 KO mice exhibited significantly shorter retention latencies in the passive avoidance test compared to WT mice, indicating long‐term memory deficits (Figure 2e,f). Similarly, TRPA1 KO mice showed reduced exploration of displaced objects in the object place recognition test, indicative of spatial memory impairments (Figure 2g,h). These results suggest that TRPA1‐dependent astrocytic calcium signaling is associated with synaptic plasticity and memory‐related processes. The reduction of astrocytic calcium signaling observed in TRPA1 knockout mice coincides with deficits in LTP, supporting a role for TRPA1‐dependent astrocytic Ca^2+^ signaling in plasticity‐related astrocyte‐neuron interactions.

To determine whether the impairment in synaptic plasticity observed in TREK‐1 and TRPA1‐deficient mice was associated with alterations in astrocytic volume transients, we next examined activity‐dependent IOS in hippocampal CA1 slices. Normalized IOS recordings following TBS revealed a robust increase in light transmittance in scrambled control slices, reflecting neuronal activity–induced astrocytic volume changes (Figure 2i,k). TREK‐1 knockdown reduced the peak IOS amplitude, consistent with impaired potassium‐ and water‐dependent astrocytic swelling. In contrast, TRPA1 knockout did not significantly alter the magnitude of the IOS response relative to scrambled controls, indicating that TRPA1 is not required for the generation of astrocytic volume transients (Figure 2i,k,m). To further assess whether TREK‐1 or TRPA1 deletion influenced the temporal properties of astrocytic volume dynamics beyond peak amplitudes, IOS traces were peak‐scaled to enable direct comparison of rise and decay kinetics independent of amplitude differences. Peak‐scaled analyses over the full 10‐min recording period (Figure 2j) and during the initial 60 s following TBS (Figure 2l) revealed comparable IOS kinetics between scrambled control, shTREK‐1 and TRPA1 knockout slices. Quantitative analysis of IOS decay time constants (tau), either normalized to baseline or following peak‐scaling, showed no significant differences between groups (Figure 2n,o). These findings indicate that TREK‐1, and not TRPA1, is associated with astrocytic volume transient initiation. Given that TRPA1 channels are known to mediate calcium influx in astrocytes (Woo et al. 2020), we next directly assessed astrocytic calcium signaling following neuronal stimulation. Using an astrocyte membrane‐targeted calcium indicator (AAV9‐GFAP‐LCK‐GCaMP6f), we observed robust stimulus‐evoked calcium elevations in TRPA1 WT astrocytes following TBS (Figure 2p). In contrast, TRPA1 KO astrocytes exhibited significantly attenuated calcium responses, despite intact IOS dynamics. Quantification of peak calcium signals confirmed a reduction in calcium elevations in TRPA1 KO astrocytes compared to WT controls (Figure 2q). Together, these findings support a model in which TREK‐1 contributes to activity‐dependent astrocytic swelling, whereas TRPA1‐dependent signaling is more closely associated with downstream astrocytic calcium responses than with the initiation of volume transients themselves.

### 3.3 Astrocytic Calcium and BDNF as Determinants of Synaptic Plasticity and Memory

**FIGURE 3:**
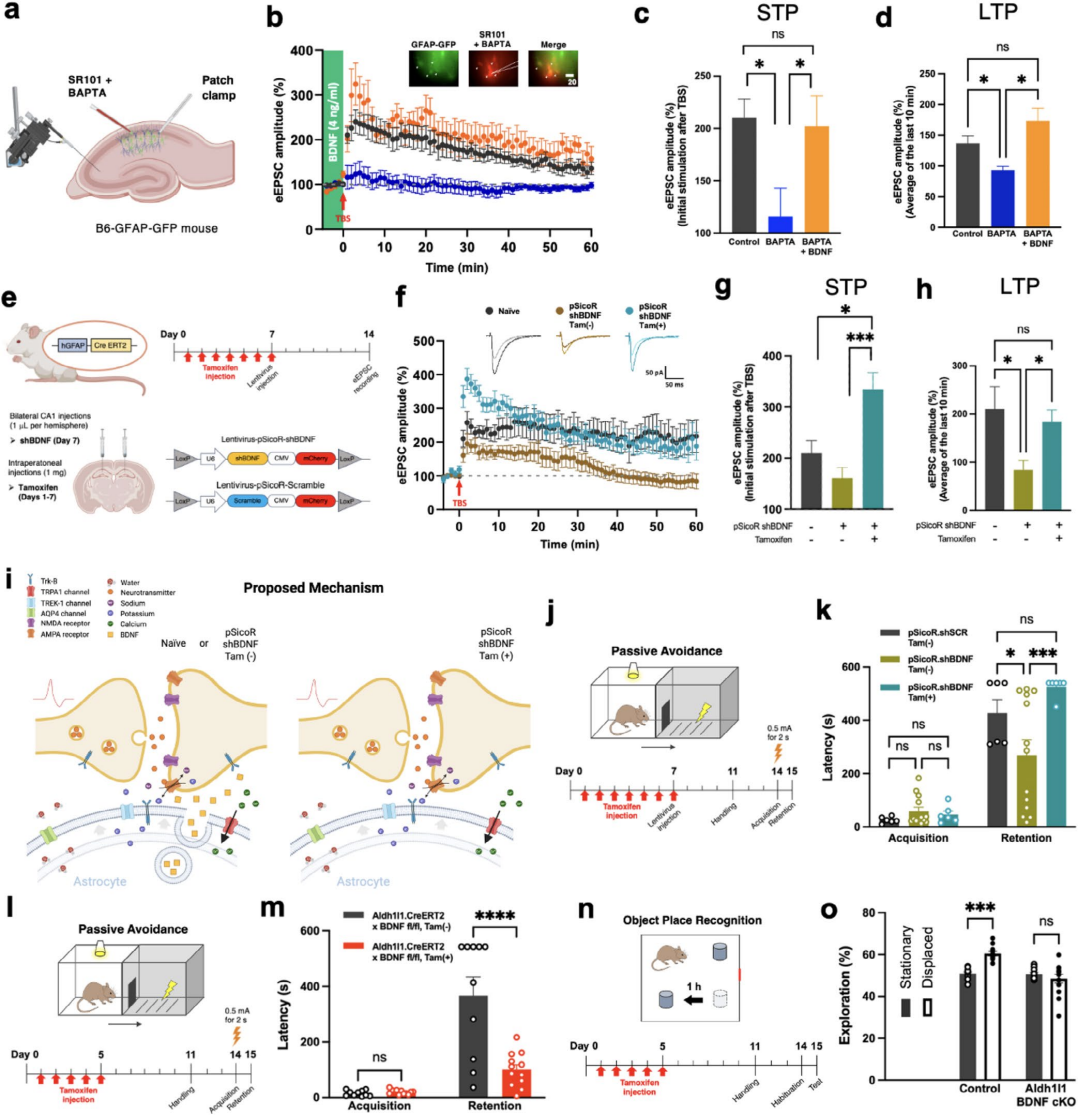
Astrocytic BDNF supports synaptic plasticity and memory by modulating calcium dynamics. (a) Schematic of hippocampal slice preparation and treatment with BAPTA and SR101. Astrocytes were loaded with BAPTA to chelate intracellular calcium, and SR101 dye was used for astrocyte visualization. (b) Time‐course of eEPSC amplitudes following TBS in hippocampal slices. Control slices show robust LTP, while BAPTA‐treated slices exhibit impaired LTP. Supplementing BAPTA‐treated slices with BDNF restored LTP. Insets show astrocytic SR101 labeling and BAPTA loading. (c, d) Quantification of eEPSC amplitudes immediately after TBS (c) and during the final 10 min of recording (d). BAPTA treatment reduces synaptic potentiation, which is rescued by BDNF supplementation. Statistical comparisons for panels (c) and (d): One‐way ANOVA with Tukey post hoc test (*p < 0.05; ns = not significant). Data are presented as mean ± SEM. Control, *n* = 9; BAPTA, *n* = 7; BAPTA+BDNF, *n* = 6. (e) Experimental timeline for astrocyte‐specific knockdown and rescue of BDNF. Mice received bilateral CA1 injections of lentivirus encoding shBDNF or scrambled control. Astrocytic BDNF expression was restored via tamoxifen treatment. (f) eEPSC amplitude time‐course following TBS in naïve, shBDNF, and tamoxifen‐treated shBDNF mice. BDNF knockdown impairs LTP, while tamoxifen‐mediated rescue restores normal synaptic plasticity. Representative traces are shown above the graph. Naïve, *n* = 4; shBDNF Tam(−), *n* = 8; shBDNF Tam(+), *n* = 6. (g, h) Quantification of eEPSC amplitudes immediately after TBS (g) and during the final 10 min of recording (h). Statistical analysis confirms significant rescue of LTP in tamoxifen‐treated shBDNF mice (*p < 0.05; **p < 0.01; ns = not significant). Statistical comparisons for panels (g) and (h): One‐way ANOVA with Tukey post hoc test (*p < 0.05; ***p < 0.001; ns = not significant). Data are presented as mean ± SEM. (i) Proposed model illustrating a potential role of astrocytic BDNF in synaptic plasticity. Calcium influx through TRPA1 channels in astrocytes is associated with BDNF release, enhancing presynaptic neurotransmitter release. In BDNF‐deficient astrocytes, this pathway is disrupted, impairing synaptic efficacy and plasticity. (j–o) Behavioral assessments of BDNF function in memory. (j, l) Passive avoidance task timelines. (k) shBDNF mice exhibited a decreased retention latency, consistent with impaired long‐term memory consolidation. shSCR, *n* = 6; shBDNF Tam(−), *n* = 13; shBDNF Tam(+), *n* = 6. (l, m) BDNF knockdown reduces retention latency, reflecting deficits in long‐term memory, which are rescued by tamoxifen‐mediated restoration of astrocytic BDNF. ALDH1L1|1.CreERT2 × BDNF fl/fl, Tam(−), *n* = 10; ALDH1L1|1.CreERT2 × BDNF fl/fl, Tam(+), *n* = 12. (n) Object‐place recognition test timeline. (o) Exploration ratios during object‐place recognition. BDNF knockdown impairs spatial memory, as indicated by reduced exploration of displaced objects. Tamoxifen treatment restored performance. Control, *n* = 9; ALDH1L1|1 BDNF cKO, *n* = 10. Statistical comparisons for panels (k), (m), and (o): Two‐way ANOVA with Bonferroni post hoc test (*p < 0.05; ***p < 0.001; ****p < 0.0001; ns = not significant). Data are presented as mean ± SEM.

To elucidate the role of astrocytic signaling in synaptic plasticity, we adopted the necessity and sufficiency experimental framework, modeled after the approach used in a previous study (Henneberger et al. 2010), where astrocytic calcium was clamped, and plasticity was subsequently rescued with a gliotransmitter. Following this paradigm, we sought to demonstrate that astrocytic calcium signaling is necessary for synaptic plasticity. To establish necessity, we utilized B6‐GFAP‐GFP mice which express a mutant form of GFP (hGFP‐S65T) under the control of the hGFAP promoter. Astrocytes within the CA1 of hippocampal slices from B6‐GFAP‐GFP mice were patched with micropipettes loaded with the high‐affinity calcium chelator BAPTA and SR101, a red fluorescent xanthene dye that diffuses through gap junctions (Figure 3a). SR101 diffusion enabled clear visualization of the astrocyte syncytium, ensuring astrocyte specificity of the calcium clamp. BAPTA sequestered intracellular calcium, effectively clamping astrocytic calcium dynamics, as previously described (Henneberger et al. 2010). In BAPTA‐loaded syncytial astrocyte slices, TBS failed to induce STP or LTP, as indicated by eEPSC amplitudes not significantly exceeding baseline value following TBS (Figure 3b–d–). These findings suggest that astrocytic volume transient‐induced calcium signaling may play a role in mediating synaptic plasticity. To assess whether astrocytic calcium signaling modulates synaptic plasticity via the release of BDNF, we examined whether BDNF alone is sufficient to restore synaptic plasticity in conditions of calcium depletion, given its well‐established roles in synaptic plasticity and memory (Crozier et al. 2008). Sufficiency was assessed by introducing exogenous BDNF into the same experimental setup (Figure 3b). Exogenous BDNF treatment in BAPTA‐treated slices fully restored STP and LTP, elevating eEPSC amplitudes during the final 10 min of recording to levels comparable to untreated controls (Figure 3c,d). These results indicate that BDNF is sufficient to compensate for the loss of calcium‐dependent astrocytic signaling.

To further explore the necessity of astrocytic BDNF, GFAP‐CreERT2 mice were utilized to conditionally silence general BDNF expression. Mice received tamoxifen injections to activate Cre, followed by bilateral CA1 lentiviral injections of shBDNF (Figure 3e). The efficacy of the shBDNF was validated in previous research (Yoo et al. 2016). In slices from these animals, TBS‐induced LTP was significantly impaired, with eEPSC amplitudes rapidly declining post‐TBS (Figure 3f). STP, however, was unaffected (Figure 3g), indicating that astrocytic BDNF specifically contributes to sustained synaptic plasticity. Restoration of astrocytic BDNF expression via tamoxifen rescue significantly increased LTP (Figure 3h), supporting astrocytic BDNF as a critical downstream effector of calcium signaling.

These results indicate that BDNF is sufficient to restore LTP under conditions of disrupted astrocytic calcium signaling, expanding on the necessity and sufficiency framework established for D‐serine in synaptic plasticity, implicating BDNF as a pivotal component of astrocytic regulation of LTP. Mechanistically, our results are consistent with the hypothesis that BDNF release is driven by calcium influx through mechanosensitive TRPA1 channel activation, triggered by astrocytic swelling during potassium and water uptake, which may enhance synaptic plasticity through pre‐, post‐, and peri‐synaptic activation of tyrosine receptor kinase B (Trk‐B) (Figure 3i).

To assess the impact of astrocytic BDNF deficiency on memory, we performed passive avoidance (Figure 3j–m) and object place recognition tests (Figure 3n,o). In the passive avoidance task, we used GFAP‐CreERT2 transgenic mice to induce general BDNF gene‐silencing or rescue with tamoxifen (Figure 3j). These behavioral assays revealed significant deficits in both spatial and long‐term memory. Mice lacking BDNF exhibited reduced retention latency, indicative of impaired long‐term memory consolidation (Figure 3k). Astrocytic BDNF rescue via tamoxifen administration restored retention latency to durations similar to controls. To further explore whether these effects extended beyond the GFAP‐positive astrocyte population, as well as to determine the effects of astrocyte‐specific gene‐silencing of BDNF, we utilized ALDH1L1‐CreERT2 x BDNF fl/fl mice, which target a broader subset of astrocytes (Figure 3m) (Liu et al. 2022; Cahoy et al. 2008). Tamoxifen‐mediated removal of astrocytic BDNF led to significant retention deficits, demonstrating the necessity of astrocytic BDNF in spatial memory (Figure 3l,m). The object place recognition test was utilized to further validate the necessity of BDNF for long‐term memory (Figure 3n). In control mice, a greater proportion of time was spent exploring displaced objects, reflecting intact spatial memory (Figure 3o). In contrast, ALDH1L1‐CreERT2 × BDNF fl/fl mice with tamoxifen‐induced astrocytic BDNF deficiency showed impaired spatial memory, as indicated by diminished exploration of displaced objects. Together, these findings suggest an integral role of astrocytic BDNF in synaptic plasticity and memory processes, possibly mediated through astrocytic volume transient‐ and calcium‐dependent mechanisms.

## 4 Discussion

This study investigates the potential role of astrocytic volume transients in regulating synaptic plasticity and memory formation, with TREK‐1‐mediated potassium buffering, TRPA1‐dependent calcium signaling, and astrocytic BDNF release emerging as key components of this process. By integrating these findings with prior research (Woo et al. 2020; Woo et al. 2019; Henneberger et al. 2010), we provide a cohesive framework highlighting astrocytic contributions to synaptic plasticity and cognitive functions. We propose a comprehensive model in which (1) astrocytic TREK‐1 channels initiate potassium buffering and volume transients, (2) TRPA1 channels convert these mechanical signals into calcium influx, and (3) the resulting astrocytic release of BDNF enhances synaptic plasticity. The dynamic interplay between synaptic activity and astrocytic volume transients suggests a role for astrocytes in synaptic plasticity. Our observations of synchronous increases in IOS changes and normalized eEPSC amplitudes following TBS (Figure 1B) illustrate the precise coupling between astrocytic volumetric responses and synaptic plasticity. The rapid rise and subsequent normalization of IOS signals, mirroring the decay kinetics of STP, suggest that astrocytic volume transients actively contribute to synaptic potentiation. This mechanism facilitates sustained elevation in eEPSC amplitudes that stabilize at over 50% of the baseline levels during LTP.

The use of IOS imaging to infer astrocytic volume transients is supported by both foundational and more recent experimental work. Early studies by MacVicar and Hochman (1991) demonstrated that IOS signals in hippocampal slices are driven by synaptically evoked cellular swelling, are abolished by blockade of excitatory transmission, and are reduced by manipulations known to suppress astrocytic osmotic swelling, identifying astrocytes in the stratum radiatum as the most plausible cellular source (MacVicar and Hochman 1991). More recently, this interpretation has been directly validated by Woo, Kim, et al. (2018), who combined IOS imaging with single‐cell confocal volume measurements to show that neuronal activity‐evoked IOS signals correspond specifically to astrocytic, rather than neuronal, volume changes. In that study, astrocyte‐specific knockdown of AQP4 almost entirely abolished IOS signals and eliminated astrocytic volume transients while leaving neuronal volume unchanged, establishing IOS as a reliable readout of astrocytic swelling under physiological stimulation conditions. Here, we focus on the relative change in transmittance (Δ*T*/*T*) and its spatiotemporal dynamics, rapid onset following TBS and decay on the timescale of short‐term potentiation, which closely mirror the astrocytic volume transients described previously. Consistent with these studies, we show that IOS signals are selectively attenuated by perturbations that disrupt astrocytic volume regulation, including TREK‐1 knockdown, while remaining intact under conditions that alter astrocytic Ca^2+^ signaling without affecting volume dynamics. Although IOS does not provide cell‐type‐specific measurements on its own, historical evidence combined with direct volumetric validation, and perturbation‐specific sensitivity supports its use here as a physiologically grounded method to monitor activity‐dependent astrocytic volume transients in real time. Our findings demonstrate that TREK‐1 plays a pivotal role in modulating both STP and LTP, as evidenced by significant impairments in these processes following TREK‐1 gene‐silencing, accompanied by deficits in spatial and long‐term memory tasks, underscoring the importance of TREK‐1 in astrocytic volume dynamics and, consequently, synaptic plasticity and memory.

In contrast to TREK‐1, our data indicate that TRPA1 channels, while essential for LTP, do not influence STP, suggesting a TREK‐1‐specific mechanism in the initial phases of synaptic potentiation. Recent findings by Woo et al. provide compelling support for this conclusion by identifying TREK‐1 as a mediator of fast, calcium‐independent glutamate release from astrocytes (Woo et al. 2012). This mode of release is initiated through activation of G_αi_‐protein coupled receptor signaling and the dissociation of G_βγ_ subunits, which directly interact with TREK‐1 to open the channel and promote glutamate release (Woo et al. 2012). It is possible that this rapid glutamate release could target metabotropic glutamate receptors facilitating transient synaptic enhancement characteristic of STP. However, this possibility has not been tested in this study. Because TREK‐1‐mediated release of glutamate requires activation of G_αi_‐protein coupled receptor signaling, the TREK‐1 mediated volume transients may not involve glutamate release through TREK‐1. These possibilities require future investigations.

Our previous research has demonstrated that astrocytic K^+^ influx through TREK‐1 channels is associated with membrane swelling, which precedes the mechanosensitive Ca^2+^ channel TRPA1. This TRPA1‐mediated Ca^2+^ influx was shown to be involved in the activation of the Ca^2+^‐dependent Cl^−^ channel Best1, and occurs independently of IP_3_R2, placing TRPA1 downstream of TREK‐1 in the signaling cascade linking astrocytic swelling to calcium‐dependent gliotransmission (Woo et al. 2020). In our present study, our findings demonstrate that TRPA1 deficiency is associated with impaired LTP without affecting STP, suggesting that TRPA1‐dependent calcium signaling may contribute preferentially to later phases of synaptic potentiation. Similarly, BDNF gene‐silencing disrupts LTP while sparing STP, further supporting that STP is regulated by mechanisms independent of TRPA1‐mediated astrocytic calcium signaling. These findings, distinct mechanisms for STP and LTP, emphasize the role of astrocytic volume dynamics in regulating STP. Recent work conducted by Ucar et al. (2021) and Kasai et al. (2023) attribute STP to mechanical transmission initiated by post‐synaptic spine swelling following neuronal activity, which causes bumping into the presynaptic neuron, thereby increasing presynaptic neurotransmitter release through enhanced SNARE assembly (Kasai et al. 2023; Ucar et al. 2021). Based on our study, we propose an alternative in which astrocytic volume transients may underlie mechanical transmission in STP through astrocytic mechanical interactions with the pre‐synaptic membrane. This proposed idea is supported by our findings that TREK‐1 gene‐silencing, known to impair astrocytic volume changes (Woo et al. 2020), reduces STP, while TREK‐1 restoration rescues it. This possibility warrants further investigation.

The foundational work by Henneberger et al. demonstrated that astrocytic calcium signaling is necessary for synaptic plasticity, largely through the release of D‐serine as a gliotransmitter to activate NMDA receptor co‐agonist sites (Henneberger et al. 2010). While this work underscored D‐serine's importance in LTP, emerging evidence (Chen et al. 2021; Koh et al. 2022), including findings from our investigation, suggests that astrocytic BDNF serves as a more versatile and potent modulator of LTP. Recent research, such as that of Koh et al. demonstrated that astrocytic D‐serine contributes predominantly to heterosynaptic long‐term depression (LTD), a process integral to cognitive flexibility (Koh et al. 2022). These findings, while highlighting D‐serine's role in synaptic plasticity, reveal its relatively specialized function in heterosynaptic LTD but not in homosynaptic LTP, contrasting with the broader and multifaceted effects of astrocytic BDNF. Unlike D‐serine, which acts primarily by enhancing post‐synaptic NMDAR activity, astrocytic BDNF exerts influence across pre‐, post‐, and peri‐synaptic membranes via TrkB receptors (Crozier et al. 2008; Song 2024; Vignoli et al. 2016).

Our study demonstrates that BDNF is both necessary for LTP induction and sufficient for LTP restoration under conditions of disrupted astrocytic calcium dynamics, as evidenced by experiments involving BAPTA‐mediated calcium clamping and targeted astrocytic gene‐silencing. The ability of exogenous BDNF to rescue LTP in BAPTA‐loaded slices suggests that calcium‐dependent astrocytic signaling contributes to the regulation of synaptic plasticity. Given that TRPA1 channels are known to mediate BDNF release through swelling‐induced calcium influx (Woo et al. 2020), it is reasonable to infer that the BAPTA‐sensitive calcium signal is mechanosensitive in nature and downstream of astrocytic volume transients. However, future studies incorporating direct measurements of extracellular BDNF dynamics, TrkB phosphorylation states, or pharmacological rescue experiments following acute TRPA1 inhibition will be important for determining whether TRPA1‐dependent astrocytic calcium signaling directly regulates BDNF release during LTP. Although our study focused on TRPA1‐associated Ca^2+^ influx in response to astrocytic volume transients, the BAPTA‐mediated calcium clamp used in this study would broadly affect multiple astrocytic calcium signaling pathways, including intracellular store‐mediated Ca^2+^ release via IP_3_ receptors. It is also worth noting that while TRPA1 knockout and TREK‐1 knockdown are not expected to directly disrupt IP_3_ signaling, indirect effects on endoplasmic reticulum Ca^2+^ dynamics, membrane potential, or metabolic tone could influence store‐mediated responses. Thus, while our findings support a TRPA1‐BDNF mechanism as a primary route, we cannot exclude the possibility that IP_3_‐dependent Ca^2+^ signaling is secondarily affected. Future studies using compartment‐specific Ca^2+^ indicators could help resolve this distinction.

Importantly, behavioral assays revealed significant impairments in memory and plasticity following astrocyte‐specific BDNF gene‐silencing, further supporting its critical role in spatial and long‐term memory. The work of Koh et al. reinforces our findings by elucidating the mechanism by which astrocytic D‐serine release regulates NMDAR tone and LTD (Koh et al. 2022). However, their data also underscore the limitations of D‐serine as a modulator of plasticity, given its confined role in regulating NMDAR‐dependent LTD rather than LTP. Collectively, these results suggest that astrocytic BDNF, with its capacity to modulate diverse synaptic targets and restore LTP under impaired calcium signaling, represents a more comprehensive gliotransmitter for LTP than D‐serine.

While numerous studies suggest the necessity of neuronal BDNF in hippocampal LTP, particularly at the Schaffer collateral‐CA1 synapse (Patterson et al. 1996; Harward et al. 2016; Lin et al. 2018; Zakharenko et al. 2003), these findings do not preclude the existence of additional, cell‐type‐specific sources of BDNF that modulate synaptic plasticity in parallel, perhaps even to a greater extent. Recent work has highlighted autocrine BDNF–TrkB signaling within single dendritic spines as a rapid and spine‐localized mechanism for LTP induction (Harward et al. 2016), while Lin et al. (2018) demonstrated that presynaptic and postsynaptic pools of neuronal BDNF differentially contribute to the induction versus maintenance phases of LTP. Our findings propose a novel, complementary mechanism whereby astrocytic BDNF contributes to LTP, potentially by acting on extrasynaptic or perisynaptic TrkB receptors in a paracrine fashion, distinct from the tightly localized autocrine neuronal mechanism.

One possible explanation for the astrocytic contribution is that astrocytes, through their extensive encapsulation of synapses and activity‐dependent volume transients, may release BDNF in response to neuronal activity in a manner that supports or stabilizes late‐phase LTP, especially under conditions where neuronal BDNF is insufficient or spatially restricted. This idea aligns with emerging models in which astrocytes act as integrators of synaptic activity, releasing gliotransmitters, including BDNF, that modulate neuronal excitability and plasticity over broader spatiotemporal scales (Liu et al. 2022). Thus, rather than replacing neuronal BDNF, astrocytic BDNF may serve as an amplifier or modulator, ensuring robust plasticity during heightened network demands or altered synaptic states. Additional research using astrocyte‐specific knockouts and cell‐type‐specific BDNF quantification will be beneficial to fully delineate the interplay between neuronal and astrocytic BDNF pools in shaping hippocampal plasticity.

One limitation of our study is that our shRNA approach involved global knockdown followed by astrocyte‐specific rescue, which raises the possibility that observed phenotypes reflect compensatory effects from other cell types. While our rescue data support a functional role for astrocytic TREK‐1 and BDNF, they do not definitively establish cell‐type‐specific necessity. Future studies using astrocyte‐specific knockdown strategies, such as DIO‐flanked shRNAs delivered into GFAP‐CreERT2 mice, could test astrocyte‐specific roles more directly. Complementary rescue in neurons (e.g., via CaMKII‐Cre) may also help determine whether astrocytic expression is uniquely required. Furthermore, while our findings highlight the roles of TREK‐1‐mediated astrocytic volume transients and TRPA1‐dependent BDNF release in synaptic plasticity, future studies leveraging advanced optogenetic tools, such as Opto‐Stim1 (Kwak et al. 2020), could enable more precise astrocyte‐specific calcium modulation, offering a system to directly investigate the interplay between astrocytic calcium signaling, BDNF release, and memory. In addition, while we successfully identified behavioral deficits linked to impaired LTP in long‐term memory tasks, we did not explore the functional implications of impaired STP on short‐term memory. Implementation of short‐term memory tasks, such as Y‐maze alternation and brief‐interval novel object recognition, could shed light on the specific contributions of astrocytic volume transients to short‐term memory.

In conclusion, this study supports a role for astrocytic volume transients as integral components of synaptic plasticity and memory. By elucidating the roles of TREK‐1, TRPA1, and BDNF, we provide a comprehensive framework for understanding astrocyte‐neuron interactions via physical volume changes and their impact on cognition. These findings lay the groundwork for novel therapeutic approaches targeting astrocytic volume pathways to enhance synaptic plasticity and memory.

## Author Contributions

C. Justin Lee conceived and designed this project. Junsung Woo, Victor James Drew, Jung Moo Lee, and Joungha Won performed the experiments. Junsung Woo, Jung Moo Lee, and Victor James Drew analyzed the data. Victor James Drew wrote the manuscript with inputs from other authors. C. Justin Lee edited the manuscript.

## Funding

This work was supported by the IBS‐R001‐D2.

## Ethics Statement

All animal procedures were approved by the Institutional Animal Care and Use Committee (IACUC) of the Institute for Basic Science (IBS, Daejeon, Republic of Korea; Protocol No. IBS‐22‐26) and were conducted in accordance with institutional and national guidelines for the care and use of laboratory animals.

## Conflicts of Interest

The authors declare no conflicts of interest.

## Supporting information


**Figure S1:** Immunohistochemical validation of astrocytic TREK‐1, TRPA‐1, and BDNF perturbation in hippocampal CA1.

**Table S1:** Primary antibodies used for immunohistochemistry analyses.

**Table S2:** Statistical summary of all experimental analyses performed in Figures 1–3.

## Data Availability

The data that support the findings of this study are available on request from the corresponding author.
