# Supplementary figures and images for: A TREK‐1/AQP4/TRPA1/BDNF Signaling Axis Is Associated With Astrocytic Volume Transients, Synaptic Plasticity, and Spatial Memory
Source: Glia. 2026 Jul 15;74(9):e70195. doi: 10.1002/glia.70195 (PMC13373336; doi:10.1002/glia.70195)

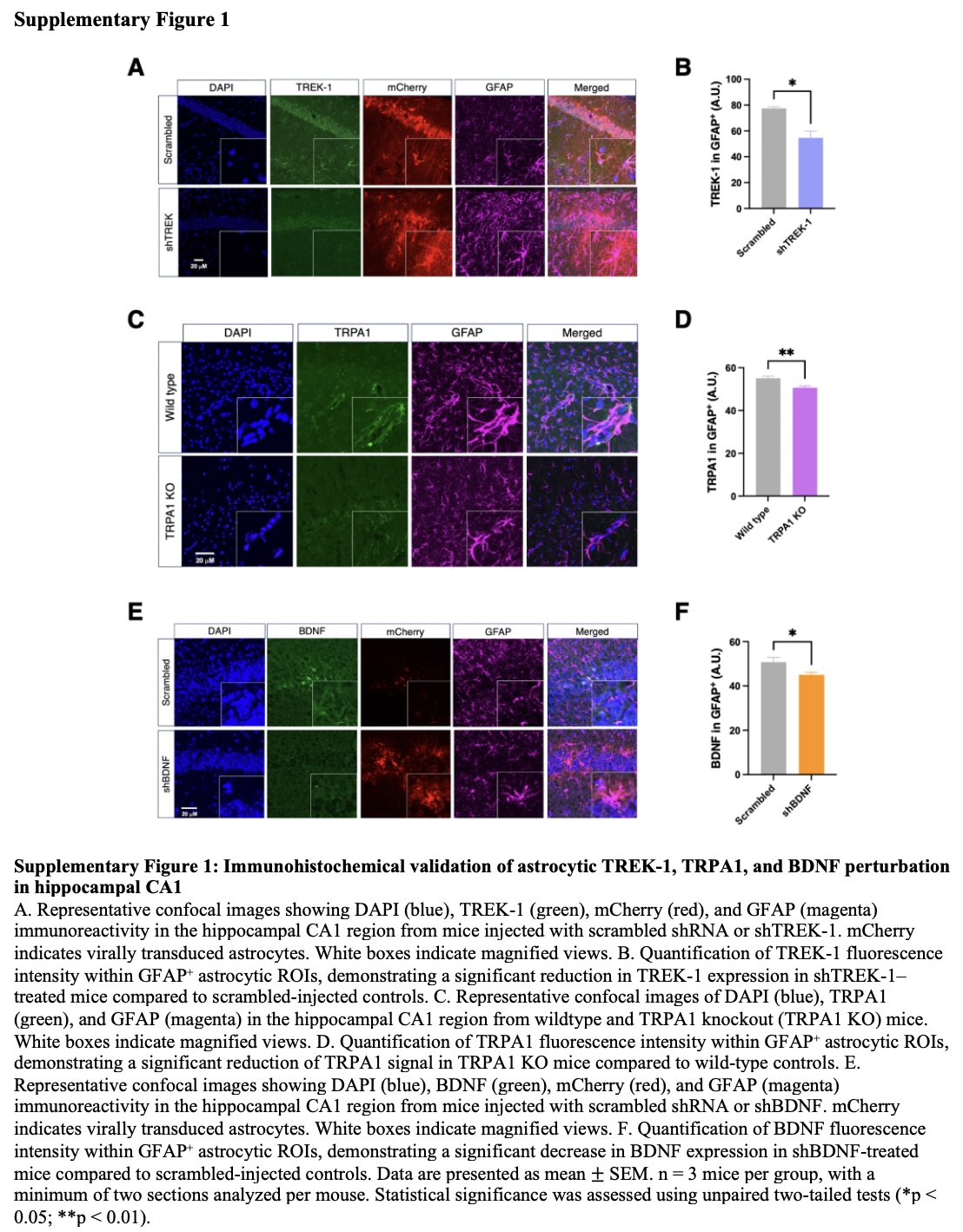

Supplement: Supplementary file 1 — Figure S1: Immunohistochemical validation of astrocytic TREK‐1, TRPA‐1, and BDNF perturbation in hippocampal CA1. [file GLIA-74-0-s003.jpg]
