# Supplementary material for: A TREK‐1/AQP4/TRPA1/BDNF Signaling Axis Is Associated With Astrocytic Volume Transients, Synaptic Plasticity, and Spatial Memory
Source: Glia. 2026 Jul 15;74(9):e70195. doi: 10.1002/glia.70195 (PMC13373336; doi:10.1002/glia.70195)
Supplement: Supplementary file 2 — Table S1: Primary antibodies used for immunohistochemistry analyses. [file GLIA-74-0-s001.pdf]

Supplementary table 1

Antibody details

Antibodies	Vendor	Catalog number	RRID	Species	Dilution	Application	Clonality
Anti-KCNK2 (TREK-1)	Alomone labs	APC-047-GP	AB_2040136	Guinea pig	1:200	IHC	Polyclonal
Anti-TRPA1/TSA	Novus Biologicals	NB110-40763	AB_715124	Rabbit	1:200	IHC	Polyclonal
Anti-BDNF	Santacruz	SC20981	AB_2064213	Rabbit	1:200	IHC	Polyclonal

Supplementary Table 1. Primary antibodies used for immunohistochemistry analyses.
