# Supplementary material for: A TREK‐1/AQP4/TRPA1/BDNF Signaling Axis Is Associated With Astrocytic Volume Transients, Synaptic Plasticity, and Spatial Memory
Source: Glia. 2026 Jul 15;74(9):e70195. doi: 10.1002/glia.70195 (PMC13373336; doi:10.1002/glia.70195)
Supplement: Supplementary file 3 — Table S2: Statistical summary of all experimental analyses performed in Figures 1–3. [file GLIA-74-0-s002.pdf]

Supplementary table 2

Statistical summary

	Experiment	Experimental Unit	Groups Compared	Statistical Test	Post Hoc Test	Assumption Checks / Notes
Figure 1	STP quantification of eEPSC amplitude	Individual recorded neuron/slice	shSCR vs shTREK-1 vs shTREK-1+Tam	One-way ANOVA	Tukey	Normality of residuals and homogeneity of variance were assumed; observations were treated as independent experimental units.
	Final 10 min LTP quantification of eEPSC amplitude	Individual recorded neuron/slice	shSCR vs shTREK-1 vs shTREK-1+Tam	One-way ANOVA	Tukey	Normality of residuals and homogeneity of variance were assumed; observations were treated as independent experimental units.
	Passive avoidance test	Individual mouse	shSCR vs shTREK-1 vs shTREK-1+Tam	Two-way repeated measures ANOVA	Bonferroni	Normality of residuals was assumed; repeated measurements were matched within the same animal. Independence was assumed between animals.
	Object-place recognition test	Individual mouse	shSCR vs shTREK-1 vs shTREK-1+Tam	Two-way repeated measures ANOVA	Bonferroni	Normality of residuals was assumed; repeated measurements were matched within the same animal. Independence was assumed between animals.
Figure 2	STP quantification of eEPSC amplitude	Individual recorded neuron/slice	WT vs TRPA1 KO	Unpaired two-tailed Student's t-test	N/A	Normality and equal variance between groups were assumed; observations were treated as independent experimental units.
	Final 10 min LTP quantification of eEPSC amplitude	Individual recorded neuron/slice	WT vs TRPA1 KO	Unpaired two-tailed Student's t-test	N/A	Normality and equal variance between groups were assumed; observations were treated as independent experimental units.
	Passive avoidance test	Individual mouse	WT vs TRPA1 KO	Two-way repeated measures ANOVA	Bonferroni	Normality of residuals was assumed; repeated measurements were matched within the same animal. Independence was assumed between animals.
	Object-place recognition test	Individual mouse	WT vs TRPA1 KO	Two-way repeated measures ANOVA	Bonferroni	Normality of residuals was assumed; repeated measurements were matched within the same animal. Independence was assumed between animals.
	Peak IOS amplitude	Individual slice	WT vs shTREK-1 vs TRP1 KO	One-way ANOVA	Tukey	Normality of residuals and homogeneity of variance were assumed; observations were treated as independent experimental units.
	IOS decay tau (baseline normalized)	Individual slice	WT vs shTREK-1 vs TRP1 KO	One-way ANOVA	Tukey	Normality of residuals and homogeneity of variance were assumed; observations were treated as independent experimental units.
	IOS decay tau (peak-scaled)	Individual slice	WT vs shTREK-1 vs TRP1 KO	One-way ANOVA	Tukey	Normality of residuals and homogeneity of variance were assumed; observations were treated as independent experimental units.
	Peak calcium response	Individual astrocyte / imaging field	WT vs TRPA1 KO	Unpaired two-tailed Student's t-test	N/A	Normality and equal variance between groups were assumed; observations were treated as independent experimental units.
Figure 3	STP quantification of eEPSC amplitude	Individual recorded neuron/slice	WT vs BAPTA vs BAPTA + BDNF	One-way ANOVA	Tukey	Normality of residuals and homogeneity of variance were assumed; observations were treated as independent experimental units.
	Final 10 min LTP quantification of eEPSC amplitude	Individual recorded neuron/slice	WT vs BAPTA vs BAPTA + BDNF	One-way ANOVA	Tukey	Normality of residuals and homogeneity of variance were assumed; observations were treated as independent experimental units.
	STP quantification of eEPSC amplitude	Individual recorded neuron/slice	shSCR vs shBDNF vs shBDNF+Tam	One-way ANOVA	Tukey	Normality of residuals and homogeneity of variance were assumed; observations were treated as independent experimental units.
	Final 10 min LTP quantification of eEPSC amplitude	Individual recorded neuron/slice	shSCR vs shBDNF vs shBDNF+Tam	One-way ANOVA	Tukey	Normality of residuals and homogeneity of variance were assumed; observations were treated as independent experimental units.
	Passive avoidance test	Individual mouse	shSCR vs shBDNF vs shBDNF+Tam	Two-way repeated measures ANOVA	Bonferroni	Normality of residuals was assumed; repeated measurements were matched within the same animal. Independence was assumed between animals.
	Passive avoidance test	Individual mouse	Tam(-) vs Tam(+)	Two-way repeated measures ANOVA	Bonferroni	Normality of residuals was assumed; repeated measurements were matched within the same animal. Independence was assumed between animals.
	Object-place recognition test	Individual mouse	Control vs Aldh1l1 BDNF cKO	Two-way repeated measures ANOVA	Bonferroni	Normality of residuals was assumed; repeated measurements were matched within the same animal. Independence was assumed between animals.

Supplementary Table 2. Statistical summary of all experimental analyses performed in Figures 1–3.
